# *Salmonella enterica* Serotype Choleraesuis Infection of the Knee and Femur in a Nonbacteremic Diabetic Patient

**DOI:** 10.1155/2013/506157

**Published:** 2013-05-28

**Authors:** Alexander M. Sy, Jagbir Sandhu, Theodore Lenox

**Affiliations:** ^1^Department of Medicine, Metropolitan Hospital Center, New York Medical College, 1901 1st Avenue New York, NY 10029, USA; ^2^Department of Pathology, Metropolitan Hospital Center, New York Medical College, New York, NY 10029, USA; ^3^Division of Infectious Diseases, Department of Medicine, Metropolitan Hospital Center, New York Medical College, New York, NY 10029, USA

## Abstract

Osteoarticular infections caused by *Salmonella* are rare. The rates of osteomyelitis and septic arthritis due to *Salmonella* are estimated to be less than 1% and 0.1%-0.2%, respectively (Kato et al., 2012). *Salmonella enterica* serotype Choleraesuis is a *nontyphoidal Salmonella*, highly pathogenic in humans, usually causing septicemic disease with little or no intestinal involvement. Serotype Choleraesuis accounts for a small percentage of published studies of *Salmonella* infections in the United States. It is not commonly reported in joint fluid and bones in contrast to serotype Enteritidis and Typhi, where a considerable number of cases have been published. Chen et al. in Taiwan found that 21% of bacteremic patients with this infection subsequently develop focal infections such as septic arthritis, pneumonia, peritonitis, and cutaneous abscess (Chen et al., 1999, Chiu et al., 2004). In contrast, our patient presented with localized osteoarticular infection with *Salmonella enterica serotype Cholerasuis*, but without evidence of bacteremia.

## 1. Case

**Figure 1:**
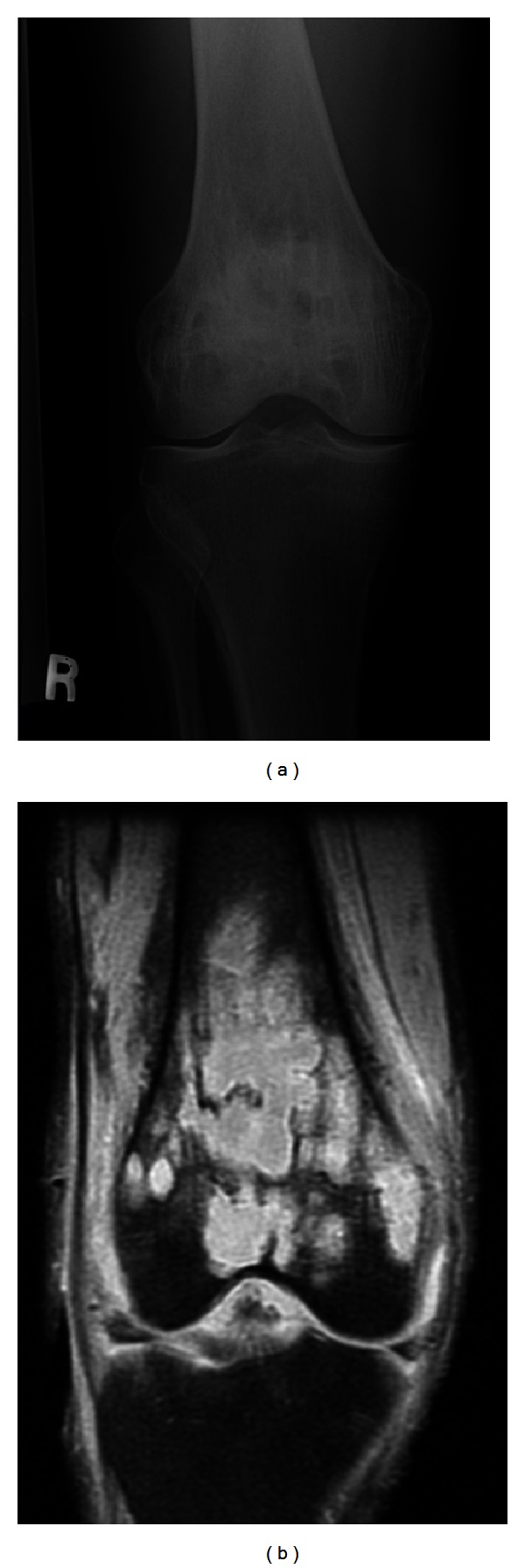
(a) and (b) showing X-ray and MRI, respectively.

**Figure 2:**
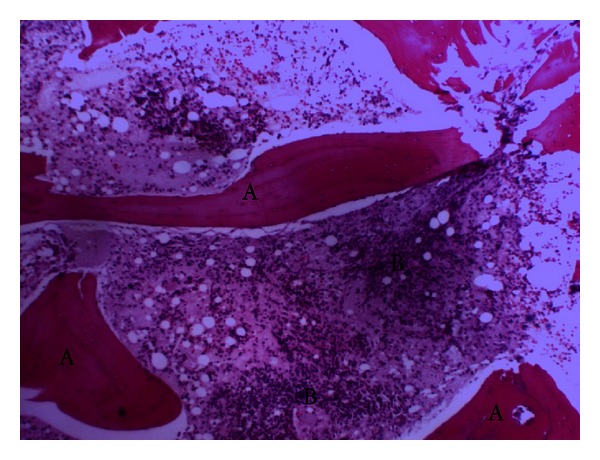
Bony trabeculae (letter A) with inflammatory cells (letter B) in bone marrow spaces.

**Figure 3:**
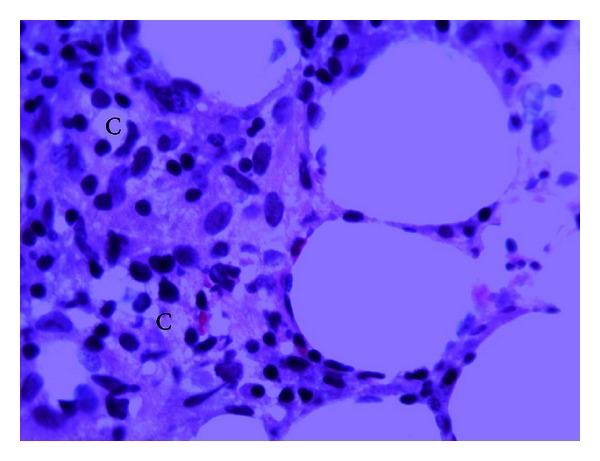
Nonnecrotizing granulomas (letter C).

**Figure 4:**
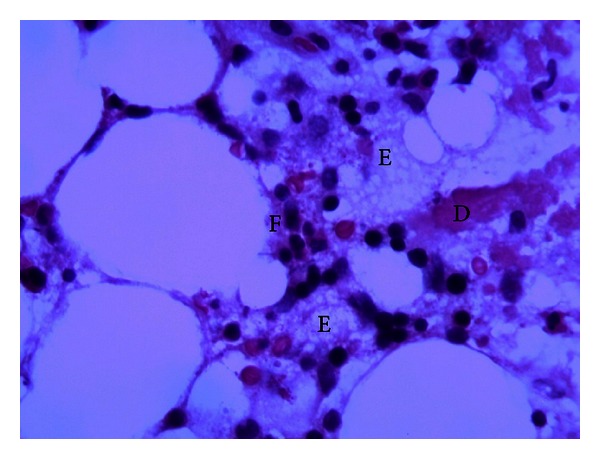
Bone necrosis (letter D), fat necrosis (letter E), and acute with chronic inflammatory cells (letter F).

A 48-year-old male from Gambia with a known history of noninsulin dependent diabetes presented with two weeks of pain and swelling over the lateral aspect of the right knee, later extending to the lateral aspect of the right thigh accompanied by fever and chills. The pain and swelling worsened so he decided to go to the emergency room. On his way, he developed five episodes of nonbloody, nonmucoid watery stools but denied nausea, vomiting, and abdominal pain. He denied use of intravenous drugs and history of trauma or surgeries. He works as a cab driver, is in a monogamous marriage, and has lived in New York City for more than ten years. He has not traveled and has not been exposed to animals or pets. Initial vitals showed blood pressure of 140/90, tachycardic at 115 beats per minute, tachypneic at 24 breaths per minute, and febrile at 38.1°C. Examination of the right lower extremity showed effusion of the knee with limitation in range of motion. There were swelling, tenderness, erythema, and fluctuance in the area of anterolateral aspect of the distal femur extending down to the anterior aspect of proximal tibia. Complete blood count revealed white blood cells of 18,000/uL with 83% polymorphonuclear cells. Glucose was 449 mg/dL, hemoglobin A1c was 12.1%, bicarbonate was 11 mmol/L, creatinine was 1.5 mg/dL, alkaline phosphatase was 265 U/L, serum ketone was large, and blood pH was 7.163. His erythrocyte sedimentation rate was 150 mm/hr, and C-reactive protein was 96.60 mg/L. The rest of the blood work-up was unremarkable. Plain radiography of the right knee showed lytic lesions at the distal femur (Figure 1(a)). Magnetic resonance imaging revealed extensive marrow abnormality of the distal femur including numerous cystic spaces, endosteal erosion, and abnormal air (Figure 1(b)). Arthrocentesis revealed a viscous turbid fluid with white blood cells of 90,000/mm^3^, and 99% was polymorphonuclear cells. Joint fluid gram stain showed no organisms. Blood, stool, and synovial fluid cultures were taken. He was admitted for septic arthritis with possible osteomyelitis and diabetic ketoacidosis. Insulin drip, intravenous fluid, and ampicillin-sulbactam were started. He was referred to orthopedic surgery where incision and drainage of the thigh and knee with manual debridement were performed. Postoperatively, the antibiotic was switched to ceftriaxone and vancomycin. His ketoacidosis improved on the second day, and intravenous insulin was shifted to subcutaneous injection. On the fifth day, blood and stool cultures were sterile, but the synovial culture came back revealing *Salmonella enterica* serotype Choleraesuis resistant to ampicillin but sensitive to chloramphenicol, trimethoprim-sulfamethoxazole, ceftriaxone, and ciprofloxacin. Hemoglobin electrophoresis was performed to rule out sickle cell disease which showed AA pattern. Patient remained febrile (38.6°C) despite maximum antibiotic therapy. Upon consultation with an infectious disease specialist, the vancomycin was discontinued, and ciprofloxacin was added with the ceftriaxone. Incision and drainage of the thigh and knee were performed four more times with femur bone biopsy, revealing chronic inflammation and focal fat necrosis of bone marrow spaces with occasional poorly formed nonnecrotizing granulomas and a few necrotic bony trabeculae (Figures 2, 3, and 4), consistent with necrotizing osteomyelitis. No acid fast bacilli or fungi were seen. Bone culture grew *Salmonella enterica* serotype Choleraesuis as well and was negative for acid fast bacilli and fungi. Computed tomography of the abdomen and echocardiogram did not show any abscess collection. He continued to show improvement with ciprofloxacin and ceftriaxone therapy. He was transferred to subacute rehabilitation for continuation of intravenous antibiotic therapy for total of 6 weeks. After completion of antibiotic therapy, his repeat erythrocyte sedimentation rate and C-reactive protein were 18 mm/hr and 1.47 mg/L, respectively.

## 2. Discussion

*Salmonella* is a nonspore forming motile Gram-negative bacillus of the family Enterobacteriaceae that colonized a wide range of mammalian hosts. It causes a broad range of infections from an asymptomatic chronic carrier state to mild gastroenteritis and to more debilitating diseases such as enteric fever, bacteremia with or without endovascular infection, and focal metastatic infections such as abscess or osteomyelitis. Salmonellae are divided into typhoidal and nontyphoidal types. The former causes typhoid and enteric fever, and the latter usually results from improperly handled food that has been contaminated with animal or human fecal material. *Nontyphoidal* Salmonellae most commonly manifest as food-borne infections. Choleraesuis is among the *nontyphoidal Salmonella* serotypes that cause bacteremia. It is a host adapted pathogen that is commonly isolated from porcine sources causing swine paratyphoid which is highly pathogenic to humans. It is uncommon in the United States as compared to Taiwan, where it ranks second only to serotype Typhimurium as the most frequently isolated *Salmonella* serotype from human sources [1]. In the United States, a total of 158 cases of confirmed human *Salmonella enterica* serotype Choleraesuis infection were reported to the Centers for Disease Control from 1999 to 2009. In 2009 alone, a total of 40,828 cases of *Salmonella* were reported of which only 22 cases were of *Salmonella enterica* serotype Choleraesuis: ten cases were identified in North Carolina, seven in Texas, and one case each was reported in Georgia, California, Massachusetts, New York, and Wisconsin [2].

*Salmonella enterica* serotype Choleraesuis usually causes septicemic disease with little or no involvement of the intestinal tract. It regularly invades the blood stream to produce bacteremic syndrome, subsequently developing local infections such as septic arthritis, pneumonia or empyema thoracis [3], peritonitis, and cutaneous abscesses. This organism is more frequently isolated from blood than from stool or any other source. Serotype Choleraesuis accounts for a small percentage of published studies of *Salmonella* infections in the United States. It is not commonly reported in joint fluid and bones in contrast to serotype Enteritidis and Typhi [4–6] where a considerable number of cases have been published.

A retrospective analysis of patients with serotype Choleraesuis bacteremia, showed that patients with malignancy, liver cirrhosis, systemic lupus erythematosus, and previous use of corticosteroid were predisposed to this infection [1, 7]. And, a study done by Chiu et al. in a medical center in Northern Taiwan found that age greater than 60 years, diabetes mellitus, peptic ulcer disease, and gastrointestinal surgery were also risk factors. These underlying diseases impair the cellular immune mechanisms or cause rapid gastric emptying and are therefore predisposing factors in developing *Salmonella* infection [8].

The overall mortality of *nontyphoid Salmonella* reported in several previous studies was in the range of 12.2–40.6%. A study by Chen et al. showed that overall mortality rate of patients with *Salmonella enterica* serotype Choleraesuis bacteremia was 30% [9]. Thus, early diagnosis and treatment are essential in treating this invasive infection. Fluoroquinolones and ceftriaxone appear to be the standard antibiotics of choice in the United States, although increasing resistance has been seen with fluoroquinolones in studies done in Taiwan [8, 9].

## 3. Conclusion

We presented a rare case of *Salmonella enterica* serotype Choleraesuis infecting the knee and adjacent bone but with no evidence of bacteremia that was successfully treated with antimicrobial and surgical interventions. This case is being reported to highlight an unusual organism with an unusual presentation. Apart from surgical intervention, definitive early diagnosis of the infection and institution of appropriate antibiotics can minimize the damage to the affected joint and may prevent mortality from this aggressive organism.
